# AIGC affordance and student self-regulation in private undergraduate education: a serial mediation model of self-efficacy and learning motivation

**DOI:** 10.3389/fpsyg.2026.1800950

**Published:** 2026-09-11

**Authors:** Shi-Zhu Liang, Zhi-Cheng Li, Yu-Ming Hsu, Jia-Lu Xu

**Affiliations:** 1 School of Business Administration, Guangzhou Institute of Science and Technology, Guangzhou, China; 2 School of Business Administration, Fu Jen Catholic University, New Taipei, Taiwan; 3 Department of Finance and International Business, Hunan University of Science and Technology, Xiangtan, China

**Keywords:** AIGC, industry-education integration, motivation, self-efficacy, self-regulated learning, student satisfaction

## Abstract

**Introduction:**

While AIGC tools are reshaping student learning, the psychological mechanisms through which AIGC affordance translates into adaptive learning behaviors remain underexplored. Grounded in Affordance-Actualization Theory and self-regulation theory, this study examines how AIGC affordance activates self-regulated learning (SRL) through the serial mediation of self-efficacy and motivation, contextualized within industry-education integration (IEI) programs in Guangzhou Private Undergraduate.

**Methods:**

A pragmatic mixed-methods design integrated quantitative surveys (*N* = 689) with qualitative interviews (*N* = 8 instructors). PLS-SEM tested the hypothesized psychological pathways; thematic analysis elucidated implementation boundary conditions.

**Results:**

The results demonstrate that perceived AIGC affordance significantly enhances students’ AIGC self-efficacy, which in turn boosts their learning motivation and ultimately strengthens their self-regulated learning (SRL) capabilities. While the quality of AIGC assessment feedback is a strong predictor of student satisfaction, it does not directly improve SRL; its influence is channeled primarily through motivation. Complementing these findings, interview data uncovered persistent gaps in IEI implementation, including curriculum-industry misalignment, limited project diversity, and weak enterprise engagement, which contextualize the quantitative model.

**Discussion:**

By integrating SRL theory with the IEI framework, this study reveals that AIGC’s primary value lies not merely as a functional productivity tool but as a psychological catalyst. It fosters adaptive learning by building students’ confidence and motivational drive. The non-significant direct path from feedback quality to self-efficacy and from satisfaction to SRL suggests that positive experience alone is insufficient for deep learning; the activation of motivational and self-efficacy mechanisms is crucial.

**Conclusion:**

The study concludes that the successful integration of AIGC in IEI requires a dual focus: strategically addressing systemic implementation gaps at the program level, and pedagogically designing AIGC activities that explicitly target the enhancement of self-efficacy and motivation to promote self-regulation.

## 1 Introduction

Industry-education integration (IEI) aligns higher education with labor market demands through project-based professional skill development. However, the widespread use of AIGC tools (e.g., ChatGPT) brings personalized learning opportunities but risks overreliance, potentially weakening students’ self-regulated learning (SRL) (Liu et al., 2025). Although the facilitating role of self-efficacy and motivation in learning outcomes (especially SRL) has been widely confirmed, three critical research gaps remain in the new context where AIGC and IEI intersect: (1) The mechanism of how AIGC promotes SRL through specific serial mediation pathways involving self-efficacy and motivation has rarely been modeled; (2) The roles of AIGC feedback quality and student satisfaction in this psychological chain remain unclear; (3) The adjustment of IEI policy pathways in the AIGC context remains underexplored. Based on the Affordance-Actualization Theory (Strong et al., 2014) and self-regulation theory (Paulsen and Feldman, 2007), this study adopts a mixed-methods research design in universities in Guangzhou, a national pilot city for industry-education integration, to provide empirical evidence for IEI policy and teaching reform in the AIGC era.

## 2 Literature review and research hypothesis

### 2.1 Literature review

#### 2.1.1 The effectiveness of integration of industry and education

IEI has evolved from “work–study” arrangements into structured models that combine academic instruction with enterprise experience, including internships, school-run enterprises, and joint education programs (Ren et al., 2018). These approaches are widely recognized as levers for vocational education reform (Moraes et al., 2023), yet policy endorsement does not guarantee effective implementation. Institutions commonly face perfunctory partnerships, limited collaboration formats, weak enterprise participation, uneven infrastructure, time constraints, and gaps in staff support (Okeke, 2025).

The theoretical foundation of this study envelops a set of key factors that shape the efficacy of industry-education integration (IEI): students’occupational proficien- cies, entrepreneurial capabilities, hands-on industry engagement, motivation, perceptions of IEI initiatives, overall satisfaction levels, and self-efficacy, and AIGC affordance. Occupational proficiencies extend beyond technical expertise to encompass academic knowledge and applied competencies critical for workplace performance. Entrepreneurial capabilities, in this context, comprise a suite of skills essential for navigating entrepreneurial ecosystems, including business planning, negotiation tactics, marketing strategies, financial literacy, and legal acumen. Hands-on industry engagement refers to direct participation in workplace settings—such as internships, apprenticeships, or entrepreneurial ventures—which has been shown to significantly shape entrepreneurial intent (Lihua, 2022). By integrating these elements, the framework seeks to holistically explain how diverse student-centered factors interact to influence the success of IEI practices.

Student perspectives are pivotal for evaluating IEI quality and its employability outcomes (Burnes, 2018). Prior work shows that hybrid learning can enhance flexibility and occupational capability (Di Marco et al., 2017), and that internships strengthen professional competence and self-efficacy (Reddan, 2016; Gosselin et al., 2021). However, students often report mismatches between curricula and workplace demands, especially under Industry 4.0 conditions (Tiron-Tudor et al., 2025). In AI-supported learning contexts, AIGC may boost motivation and learning efficiency while also increasing risks of overreliance (Liu et al., 2025; Paulsen and Feldman, 2007). These mixed findings suggest the need to examine how AIGC features translate into students’ motivation and SRL within IEI settings.

#### 2.1.2 Affordance and AIGC affordance

Gibson (1979) defined affordance as the ability to take direct action after perceiving the environment; Krancher et al. (2018) as the relationship between object features and user intentions. Grounded in affordance actualization theory (Strong et al., 2014), AIGC affordance refers to students’ perceived generative action possibilities in human-AI interaction. Unlike conventional ICT providing static information access, AIGC enables productive failure-recovery cycles: iterative prompt refinement constitutes unique mastery experiences that pre-scripted resources cannot replicate (Volkoff and Strong, 2013). Four task-activated affordances are specified: generative co-creation, conversational iteration, critical verification, and strategic redistribution of cognitive effort.

Acknowledging that affordance encompasses both latent relational potential and manifest behavioral actualization, we operationalize AIGC affordance as enacted competence—students’ perceived functional capability to deploy AIGC features in IEI task execution. This aligns with Strong et al. (2014): affordances are actualized through goal-directed action, and enacted competence indicates the degree to which perceived possibilities are integrated into performance. Because AIGC simultaneously scaffolds and risks undermining self-regulation via cognitive offloading, self-efficacy and motivation serve as gatekeepers between AIGC use and autonomous regulation.

#### 2.1.3 Student self-regulated learning

Self-regulated learning is closely associated with the process of individual knowledge construction. It refers to students’ capacity to set appropriate task-related goals, assume responsibility for their own learning, and sustain motivation throughout the learning process (Wang and Hsieh, 2022; Wei, 2023). It also involves the ability to adjust learning strategies as needed to effectively accomplish academic tasks.

Rooted in Bandura’s social cognitive theory, self-regulation is driven by internal motivation and generally comprises three phases: the forethought phase, the performance phase, and the self-reflection phase (Wang and Hsieh, 2022). Through continuous self-adjustment and goal-directed behavior, individuals progress in a cyclical manner. Self-regulated learning is also strongly linked to learning outcomes and exerts a significant influence on learning self-efficacy.

Based on individual differences, self-regulation can be categorized into internal and external regulation. Internal regulation occurs when students actively employ learning techniques such as strategic thinking and time management to facilitate knowledge acquisition. External regulation arises in response to external factors—for example, teachers’ instructional demands or assessment criteria—which prompt students to adapt their learning strategies. Importantly, self-regulation is influenced by self-confidence, often reflected in the construct of self-efficacy. Through self-regulated learning, students can control their behavior and learning goals in an active process of acquiring knowledge (Wang and Hsieh, 2022).

#### 2.1.4 Learn motivation

Motivation exerts a pivotal influence on students’ engagement with industry–education integration (IEI) programs, shaping their time allocation, persistence in learning tasks, and emotional connection to educational activities (Wang and Hsieh, 2022; Anderson et al., 2015). Strong motivation drives deeper cognitive processing and active participation in IEI practices and directly strengthens academic and skill-development outcomes (Filgona et al., 2020; Martin, 2023).

A nuanced understanding of student motivation—encompassing both intrinsic drivers (e.g., career aspirations and valuing of learning) and extrinsic factors (e.g., industry recognition and mastery orientation)—offers critical insights into students’ initial decisions to participate in IEI and their sustained commitment over time (Martin, 2023). Such insights are indispensable for refining educational strategies, designing targeted interventions, and creating supportive environments that align with students’ motivational needs, thereby enhancing the effectiveness of IEI implementation. Martin (2023) emphasizes that intrinsic motivation highlights the interplay among efficacy beliefs, meaning identification, and growth goals, and that this tripartite structure requires systematic development through instructional strategies grounded in learning-related interventions (LRI), rather than through isolated or one-off stimuli. This study therefore proposes innovative strategies for balancing pedagogical adjustments with the enhancement of students’ learning motivation.

#### 2.1.5 Satisfaction

Assessing the quality of educational initiatives relies heavily on student satisfaction, which is a critical metric that profoundly shapes learner achievement and broader perceptions of educational value. Satisfied students are more likely to complete their studies, recommend their institution, and enhance its reputation (Razinkina et al., 2018). Conceptually, it is important to distinguish between satisfaction as a cognitive–affective state and satisfaction as a behavioral manifestation. At its core, satisfaction is an outcome influenced by a constellation of factors, reflecting both the intrinsic value of the evaluated object and the perceptual lens of the evaluator (Razinkina et al., 2018; Aman et al., 2023).

In this study, satisfaction is operationalized as an evaluative construct that directly reflects the perceived quality and impact of educational service provision (Thabetha and Munyeka, 2023). Students’ satisfaction with higher education and their assessment of overall educational quality depend on their values and attitudes toward education, including their motivation for learning and the importance they attach to education and knowledge (Aman et al., 2023). Higher levels of satisfaction are associated with stronger motivational drive, greater effort investment, and more purposeful participation in academic tasks (Thabetha and Munyeka, 2023).

Within the AIGC context, satisfaction is defined as a positive response to the perceived value and experience of using the technology, and it serves as a key factor influencing continued use intention (Jo, 2023).

The theoretical model is grounded in principles of behaviorist learning theory, which underscores the significance of instructional practices and hands-on experiences in fostering student engagement and satisfaction. When effective teaching methods are paired with meaningful practical application, they boost self-efficacy—the belief in one’s ability to succeed—which in turn strengthens learning behaviors, establishing a virtuous cycle that enhances educational outcomes (Canrinus et al., 2012).

Taken together, this holistic theoretical framework elucidates the interconnected dynamics between curriculum design, practical training approaches, and the roles of educators, collectively forming a resilient infrastructure that supports the successful implementation of industry-education integration initiatives.

#### 2.1.6 AIGC affordance on students’ learning

Artificial intelligence-generated content (AIGC) is increasingly prevalent in both work and daily life, bringing students entirely new learning experiences. Researchers in the field of AIGC focus on its application in education, particularly in curriculum design, students’ willingness to use AI, and how AI technology can enhance learning outcomes (Chen et al., 2025). They explore the use of AIGC to generate personalized learning content to meet the diverse needs and interests of students, thereby increasing engagement and efficiency (Wang, C. et al., 2025; Wang, W. S. et al., 2025). Additionally, research covers the use of AIGC in assessing student performance, providing immediate feedback, and assisting teachers in decision-making. However, with the widespread integration of AIGC into the learning process, students have gradually and unconsciously come to rely on AIGC as a problem-solving assistant or an information retrieval tool to obtain answers to their inquiries. The impact of AIGC on students’ comprehension and learning outcomes is manifested in several key aspects.

#### 2.1.7 Quality of AIGC feedback

Drawing upon the DeLone and McLean (2004) Information Systems Success Model, the influence of AIGC can be fundamentally framed as a function of its information quality. The model posits that the success of any information system is predicated on the quality of the information it delivers. In the context of AIGC, information quality encompasses critical dimensions such as accuracy, relevance, timeliness, understandability, and personalization of the generated content. When AIGC exhibits high information quality—providing accurate, clear, and contextually relevant explanations—it can significantly enhance students’ learning processes. It acts as a powerful catalyst for self-regulated learning (SRL) by offering on-demand, personalized support that helps students to better monitor their understanding, identify knowledge gaps, and develop effective learning strategies. Conversely, when the information quality is poor characterized by inaccuracies (“hallucinations”), overly generic responses, or irrelevant content can inadvertently hinder deep comprehension. This over-reliance on a potentially fallible tool may lead to superficial engagement with learning materials, where students accept answers without critical evaluation, potentially undermining the development of their critical thinking and problem-solving skills.

#### 2.1.8 AIGC self-efficacy

Self-efficacy refers to an individual’s subjective assessment of their ability to finish a specific task, influence by prior experiences and self-evaluation (Poort et al., 2023). As a critical mediating variable, it impacts individual behavioral, effort exertion, and decision-making action (Wilson et al., 2020; Huang et al., 2024b). Prior research demonstrates that heightened self-efficacy positively effect on creative thinking, thereby enhancing learning outcomes (Wang et al., 2022).

Consequently, the importance of teachers’ differentiated instruction becomes clear. By tailoring teaching content and methods to students’ knowledge levels, readiness, learning styles, ages, and environmental contexts, educators can effectively enhance students’ self-efficacy and academic performance (Smale-Jacobse et al., 2019). This personalized approach not only supports student development aligned with their unique traits and abilities but also promotes improvements in critical and creative thinking skills.

### 2.2 Research hypothesis

#### 2.2.1 AIGC affordance on self-efficacy and self-regulated learning

AIGC empowers users to efficiently generate diverse content types tailored to their specific needs, including text and image generation tools, which are widely deployed across various platforms (Wu et al., 2023). This technology has made remarkable progress in the field of computer science, is penetrating into various fields and bringing about revolutionary changes, as well as reshaping the interaction dynamics between teachers and students and teaching methods (Huang et al., 2024b). AIGC has significantly enhanced students’ self-efficacy, making them more confident in their ability to complete learning tasks (Huang et al., 2024b). Wang, C. et al. (2025) and Wang, W. S. et al. (2025) point that AIGC (e.g., ChatGPT) can provide immediate, explanatory feedback and offer adjustment suggestions. This dynamic interaction significantly supports the ‘monitoring’ and ‘control’ processes of SRL, enabling students to adjust their strategies immediately rather than getting trapped in a trial-and-error cycle.

Therefore, the hypotheses as follows:

*H1a*: AIGC affordance can enhance student’s self-efficacy.

*H1b*: AIGC affordance has a positive impact on student’s self-regulated learning.

#### 2.2.2 Quality of AIGC feedback on satisfaction and self-efficacy

Wang, C. et al. (2025) and Wang, W. S. et al. (2025) found after learning, students can ask ChatGPT to help them summarize learning points, analyze error patterns, and connect new and old knowledge. It can provide precise targeted feedback and abundant learning resources, significantly enhancing the quality and efficiency of the learning experience (Liu et al., 2023). Additionally, AIGC technology enables real-time adaptation of learning content and instructional strategies based on learners’ behaviors and feedback (Wang, C. et al., 2025; Wang, W. S. et al., 2025). For example, by leveraging deep learning algorithms, generative artificial intelligence (GAI) can analyze individual learning progress and deliver personalized recommendations and guidance to assist learners in overcoming challenges and improving their problem-solving skills. Moreover, AI-powered systems can simulate realistic interactive environments, enabling learners to engage and collaborate with AI-generated avatars in virtual settings (Wang, C. et al., 2025; Wang, W. S. et al., 2025). Such interactions not only enrich the learning experience but also foster social learning opportunities, thereby enhancing learner satisfaction.

Therefore, we hypothesize:

*H2a*: Quality of AIGC feedback has a positive impact on students’ satisfaction.

*H2b*: Quality of AIGC feedback has a positive impact on students’ self-efficacy.

#### 2.2.3 Self-efficacy on learning motivation and self-regulated learning

Individuals with robust self-efficacy beliefs perceive challenging tasks as opportunities for mastery rather than insurmountable obstacles, and they demonstrate unwavering commitment to the ambitious goals they establish (Bandura, 1997). The ability of self-assessment and reflection of self-efficacy helps learners summarize learning experiences, improve future learning plans, and strengthen learning motivation (Wang, C. et al., 2025; Wang, W. S. et al., 2025).

Therefore, we hypothesize:

*H3a*: Self-efficacy has a positive impact on students’ learn motivation.

*H3b*: Self-efficacy has a positive impact on students’ self-regulated learning.

#### 2.2.4 Learn motivation and satisfaction on students’ self-regulated learning

Learn motivation and cognition are inseparable and interact with each other in the learning process (Vollmeyer and Rheinberg, 2006). It has been established that motivation influences students’ self-regulated learning (SRL) both directly and indirectly through various mediating factors. This study examines the potential impact of learning motivation on SRL within the context of AI-generated content (AIGC) environments.

Satisfaction refers to their gratification concerning system quality, format, speed, and various functionalities (Albelbisi, 2020). Zhao (2016) found that quality factors significantly influence self-regulated learning (SRL), and the relationship between satisfaction and SRL is also statistically significant.

Based on the aforementioned research findings, we hypothesize:

*H4*: Learn motivation has a positive impact on students’ self-regulated learning.

*H5*: Satisfaction has a positive impact on students’ self-regulated learning.

#### 2.2.5 Mediation hypothesis

Bandura (1997) posits that self-efficacy beliefs—shaped most powerfully by mastery experiences—serve as key cognitive mediators influencing motivational processes. This theoretical insight supports the pathway from AIGC self-efficacy to learning motivation in our model. Grounded in affordance actualization theory and self-regulation theory, we propose the following mediation hypotheses:

*H6*: Self-efficacy mediates the relationship between AFS and LM.

*H7*: Self-efficacy mediates the relationship between AFS and SRL.

*H8*: Self-efficacy mediates the relationship between QAF and LM.

*H9*: Self-efficacy mediates the relationship between QAF and SRL.

*H10*: SAT mediates the relationship between QAF and SRL.

*H11*: Self-efficacy and Learn motivation mediate the relationship between AFS and SRL.

*H12*: Self-efficacy and Learn motivation mediate the relationship between QAF and SRL.

## 3 Method

**Figure 1:**
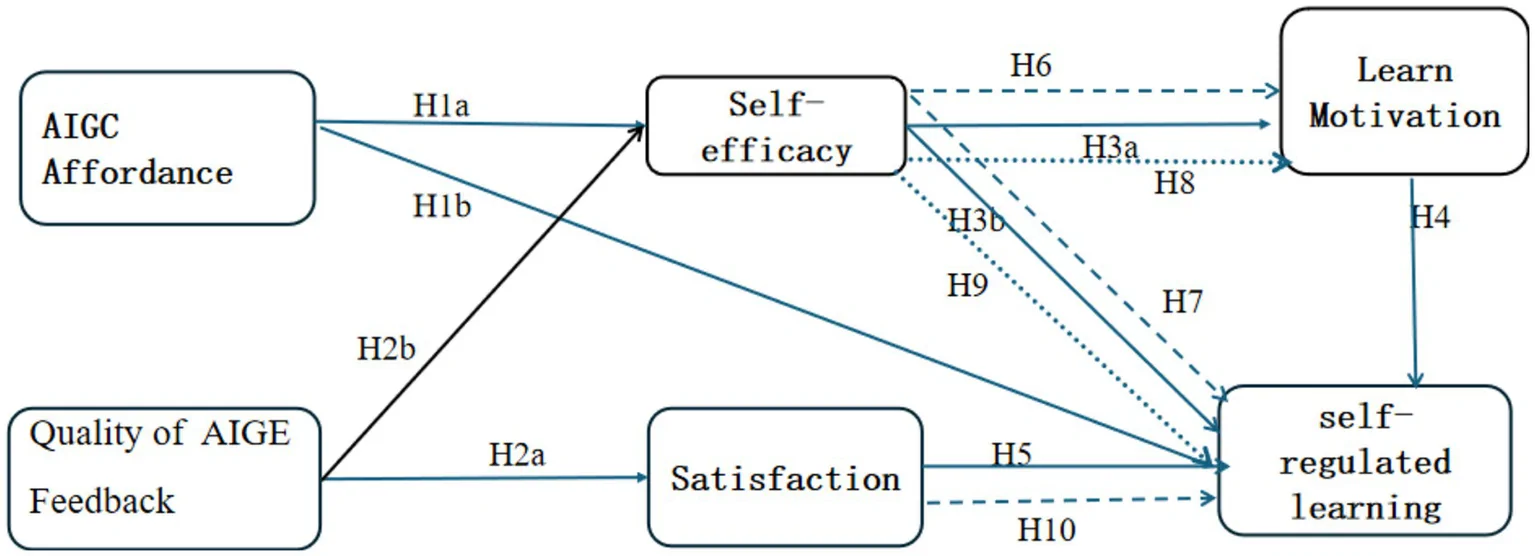
Research model.

This study adopts an interpretive sequential mixed-method design: a quantitative survey was conducted from October to November 2024 (including a 4-week IEI program, such as enterprise real-time Double Eleven marketing operation, software development tasks), and qualitative interviews were also carried out to explain the background of the non-significant path. This method combines the universality of quantitative research with the depth of qualitative research (Figure 1).

### 3.1 Construct measurement

Measurement items were adapted from previously validated studies and were modified to fit the context of the present research. Learning motivation (four items) was adapted from Qin (2021). Self-regulated learning was measured using a four-item scale adapted from Lee and Tsai (2011). Four items measuring AIGC self-efficacy were derived from Chen et al. (2025) and Wang and Chen (2024), while four items assessing the quality of AIGC assessment feedback were adapted from DeLone and McLean (2004). AIGC satisfaction (three items) was adapted from Zhang (2025). AIGC affordance (four items, operationalized as enacted competence in affordance actualization) was adapted from DeLone and McLean (2004), Volkoff and Strong (2013), and Strong et al. (2014). All items were measured using a seven-point Likert scale (1 = strongly disagree, 7 = strongly agree). Because the data were collected in China, the questionnaire was first translated into Chinese by bilingual professionals and then back-translated and reviewed to ensure semantic equivalence. The measurement items are presented in Table 1.

**Table 1:** Measurement items.

Construct	Code	Item	Adapted from
AIGC-satisfaction	SAT1	I am satisfied with my experience using AIGC technology in study.	Zhang (2025) and Spreng and Olshavsky (1993)
	SAT2	I am satisfied with the functions of AIGC technology in study.	
	SAT3	I am satisfied with the overall use of AIGC technology in study.	
AIGC affordance	AFS1	I can effectively optimize AIGC prompt words to obtain precise answers.	DeLone and McLean (2004) and Volkoff and Strong (2013) and Strong et al. (2014)
	AFS2	I can critically verify the logical loopholes in AIGC-generated content.	
	AFS3	I am accustomed to using AIGC to generate study plans and adjust their execution.	
	AFS4	The immediate feedback from AIGC makes me more willing to take on high-difficulty tasks.	
AIGC quality assessment Feedback	QAF1	I can get the answers I need from AIGC at any time.	DeLone and McLean (2004)
	QAF2	AIGC can provide targeted answers based on my questions.	
	QAF3	The explanations provided by AIGC are clear and easy to understand, which helps me better grasp the knowledge points.	
	QAF4	The feedback from AIGC is too general and lacks practical assistance.	
Learn motivation	LM1	I enjoy the process of solving complex problems in my professional courses.	Qin (2021)
	LM2	Learning new knowledge makes me excited.	
	LM3	For things that most people in society consider valuable for career selection, such as obtaining certificates and taking the CET-6 exam, I strive to acquire them.	
	LM4	I think mastering professional knowledge well will be of great help for future career selection.	
Student self-regulated learning	SRL1	I will set my own learning goals.	Lee and Tsai (2011)
	SRL2	I will adopt a student approach that suits me.	
	SRL3	I will evaluate or review my learning outcomes.	
	SRL4	I will improve my learning methods when necessary.	
AIGC self-efficacy	SEF1	I believe I can effectively utilize AIGC to answer the questions in the course.	Chen et al. (2025) and Wang and Chen (2024)
	SEF2	Even without the guidance of a teacher, I am confident that I can independently learn new knowledge through AIGC.	
	SEF3	When the first answer given by AIGC was not satisfactory, I was confident that I could obtain a better result through further questioning.	
	SEF4	I believe I can judge whether the answers generated by AIGC are right or wrong.	

#### 3.1.1 Questionnaire content

A structured questionnaire was developed to measure students’perceptions of IEI effectiveness across eight constructs, informed by prior literature and adapted to the institution’s IEI context. Key dimensions and example items (from Table 2) included:

**Table 2:** Questionnaire content.

Construct	Key focus	Example
Professional competence (PFC)	Professional competence (PFC) knowledge	PFC1: “To what extent have you acquired proficiency in your professional knowledge?” (1 = “Not at all” to 5 = “Extensively”); PFC5: “Have you participated in any competitions or projects relevant to your field of study?” (1 = “Never” to 5 = “Multiple times”)
Employability (EPA)	Employment readiness, career confidence, and entrepreneurial intentions	EPA2: “How confident are you in finding a job that meets your expectations?” (1 = “Not confident” to 5 = “Very confident”); EPA3: “Do you have an interest in starting your own business or engaging in entrepreneurial projects in the future?” (1 = “No” to 5 = “Yes, strongly”)
Practical experience (PE)	Internship/part-time job history, role impact, and skill gains	PE3: “During your internship at the base, what was the most challenging technical skill issue you encountered?” (open-ended, later coded quantitatively for frequency)
	Goal setting；self-Assessment;interest exploration	SRL1: “Establish personalized learning objectives” and SRL3: “adjust learning approaches as needed”(1 = “Strongly Disagree” to 5 = “Strongly Agree”)
Motivation	Primary drivers for IEI participation	Open-ended item: “What is your primary motivation for participating in industry-educa -tion integration initiatives?” (coded into themes: career advancement, academic require- ment, skill development, etc.)
Opinions about IEI	Opinions about IEI Perceived models, challenges, institutional service integration	OP2: “What challenges do you perceive in the education process of integrating industry and education?” (1 = “No challenges” to 5 = “Severe challenges”; follow-up open-ended: “Please specify”)
Satisfaction	Satisfaction with IEI and improvement ideas	SA1: “Please share your satisfaction level with the school’s industry-education integration project and provide any suggestions for improvement.” (1 = “Very dissatisfied” to 5 = “Very satisfied”)
Self-efficacy	Confidence in change	SE1: As long as I receive instruction from teachers or mentors, I am confident in my ability to master professional skills required for my field (1 = “Not at all” to 5 = “Extensively”)

#### 3.1.2 Sample and sampling

The student sample was purposefully selected from the institution’s undergraduate population enrolled in IEI-linked programs, with stratified random sampling applied to ensure representativeness across key demographics.

The samples were primarily drawn from undergraduate institutions that emphasize professional practice, with participants selected based on their involvement in practical projects such as internships and course-based experiential learning activities. This approach ensured sufficient statistical power for analyzing the specific outcomes of IEI. A total of 1,000 questionnaires were distributed via the institution’s Learning Management System (LMS), yielding 689 valid responses, resulting in a response rate of 68.9%. All participants were required to have used AIGC tools for at least one complete task cycle (i.e., from initial prompt to final deliverable) within their respective IEI projects, ensuring that their survey responses reflected situated, task-bound perceptions rather than general technology attitudes.

For the qualitative component, semi-structured interviews were conducted with purposively selected enterprise mentors (from manufacturing, information technology, and education) and academic instructors with >2 years IEI project supervision. This sampling maximized information richness on task authenticity while ensuring theoretical relevance to students’ project domains. Interviews were conducted in person or via Zoom, each lasting approximately 30 min.

### 3.2 Data analysis

We analysed the survey data using SmartPLS. Reliability was assessed via Cronbach’s alpha and composite reliability (≥0.70), and convergent validity via AVE (≥0.50) (Fornell and Larcker, 1981). Discriminant validity was evaluated using Fornell–Larcker and HTMT criteria, and collinearity via VIF. Structural paths and indirect effects were tested with bootstrapping (10,000 resamples) following Hair et al. (2022), reporting bias-corrected 95% confidence intervals.

## 4 Results

### 4.1 Measurement model assessment

Valuating the measurement model for this study, multiple metrics were employed to assess the reliability and validity of the constructs under investigation. Factor loadings for all items ranged from 0.891 to 0.933, well exceeding the minimum threshold of 0.7. Cronbach’s alpha values for the constructs varied from 0.895 to 0.936, and composite reliability for these constructs was measured, yielding values from 0.925 to 0.954, which also surpass the recommended threshold of 0.7. This further confirms the reliability of the constructs. Convergent validity was evaluated using the average variance extracted (AVE) for each construct, yielding values between 0.813 and 0.848—all exceeding the minimum threshold of 0.5 (Fornell and Larcker, 1981). These results confirm that a substantial portion of the variance in the indicators is explained by their respective constructs, thereby establishing strong convergent validity (Table 3).

**Table 3:** Constructs reliability and validity.

Construct	Variable	Factor loading	Cronbac’s *α*	Composite reliability	(CR) AVE
AIGC affordance	AFS1	0.897	0.923	0.946	0.813
	AFS2	0.908			
	AFS3	0.904			
	AFS4	0.897			
Learn motivation	LM1	0.891	0.925	0.947	0.817
	LM2	0.906			
	LM3	0.916			
	LM4	0.932			
AIGC quality assessment feedback	QAF1	0.921	0.910	0.943	0.848
	QAF2	0.909			
	QAF3	0.932			
Satisfaction	SAT1	0.912	0.895	0.925	0.827
	SAT2	0.895			
	SAT3	0.920			
Student self-regulated learning	SRL1	0.916	0.936	0.954	0.839
	SRL2	0.918			
	SRL3	0.904			
	SRL4	0.924			
AIGC Self-efficacy	SEF1	0.933	0.908	0.942	0.845
	SEF2	0.907			
	SEF3	0.917			

Discriminant validity was established using the Fornell-Larcker criterion, which entails comparing the square root of the average variance extracted (AVE) for each construct with the inter-construct correlations. Furthermore, the heterotrait-monotrait ratio (HTMT) served as a more rigorous test of discriminant validity, indicating a distinct separation between constructs (Tables 4, 5).

**Table 4:** Discriminant validity—Heterotrait-monotrait ratio (HTMT).

	AFS	LM	QAF	SAT	SEF	SRL
AFS						
LM	0.591					
QAF	0.848	0.713				
SAT	0.594	0.697	0.788			
SEF	0.745	0.766	0.642	0.541		
SRL	0.723	0.854	0.656	0.601	0.826	

AFS, AIGC affordance; LM, Learn Motivation; QAF, AIGC Quality Assessment Feedback; SAT, Satisfaction; SRL, Student Self-Regulated Learning; SEF, AIGC Self-efficacy.

**Table 5:** Discriminant validity—Fornell-Larcker criterion.

	AFS	LM	QAF	SAT	SEF	SRL
AFS	0.902					
LM	0.549	0.904				
QAF	0.735	0.701	0.939			
SAT	0.540	0.636	0.712	0.909		
SEF	0.684	0.702	0.584	0.490	0.919	
SRL	0.674	0.796	0.606	0.551	0.762	0.916

AFS, AIGC affordance; LM, Learn Motivation; QAF, AIGC Quality Assessment Feedback; SAT, Satisfaction; SRL, Student Self-Regulated Learning; SEF, AIGC Self-efficacy.

Finally, common method bias was assessed through multiple procedural and statistical remedies. Harman’s test (KMO = 0.934, Bartlett’s *p* < 0.001) showed the first unrotated component at 54.111%—below severe CMB levels—while varimax rotation yielded a balanced three-factor structure (ratio = 1.19). Collinearity diagnosis supplemented these results: Kock’s (2015) full collinearity VIF ranged 2.377–4.075, below the conservative 3.3 threshold (Table 6), confirming no significant collinearity issues.

**Table 6:** Collinearity diagnostic.

AFS1	2.934
AFS2	3.367
AFS3	3.294
AFS4	3.239
LM1	2.946
LM2	3.250
LM3	3.648
LM4	3.397
QAF1	3.130
QAF2	2.750
QAF3	3.469
SAT1	2.843
SAT2	2.377
SAT3	3.188
SEF1	3.472
SEF2	2.676
SEF3	3.096
SRL1	3.539
SRL2	3.786
SRL3	3.233
SRL4	4.075

AFS, AIGC affordance; LM, Learn Motivation; QAF, AIGC Quality Assessment Feedback; SAT, Satisfaction; SRL, Student Self-Regulated Learning; SEF, AIGC Self-efficacy.

### 4.2 Structural model test

The structural model was assessed using SmartPLS to examine the causal and correlational relationships among the latent variables. Model fit was evaluated using the Standardized Root Mean Square Residual (SRMR) and the Normed Fit Index (NFI). An SRMR value below 0.08 is considered acceptable. As shown in Table 7, the saturated model yielded SRMR = 0.043 and the estimated model SRMR = 0.058, both within the acceptable range. The NFI values for the saturated and estimated models were 0.862 and 0.860, respectively. Although NFI can be underestimated in smaller samples, values around 0.80 or above are generally regarded as acceptable, indicating an overall satisfactory model fit.

**Table 7:** Model fit test.

	Saturated model	Estimated model
SRMR	0.043	0.058
NIF	0.862	0.860

To obtain robust estimates of standard errors and *t*-values, we performed bootstrapping with 10,000 resamples, following the recommendations of Hair et al. (2022). The path from AIGC affordance to AIGC self-efficacy yielded a beta coefficient of 0.583 (*p* < 0.001), and the path from AIGC affordance to student self-regulated learning yielded a beta of 0.316 (*p* < 0.05), thus supporting H1a and H1b. With respect to AIGC quality assessment feedback, the path to satisfaction showed a beta of 0.712 (*p* < 0.001), supporting H2a, whereas the path to AIGC self-efficacy (*β* = 0.131, *p* > 0.05) did not support H2b.

AIGC self-efficacy exerted a substantial influence on both learning motivation (*β* = 0.565, *p* < 0.05) and student self-regulated learning (*β* = 0.250, *p* < 0.05), thereby supporting H3a and H3b. Learning motivation also had a strong positive effect on student self-regulated learning (*β* = 0.527, *p* < 0.01), supporting H4. By contrast, the path from satisfaction to student self-regulated learning (*β* = 0.032, *p* > 0.05) was not significant, and H5 was not supported.

**Figure 2:**
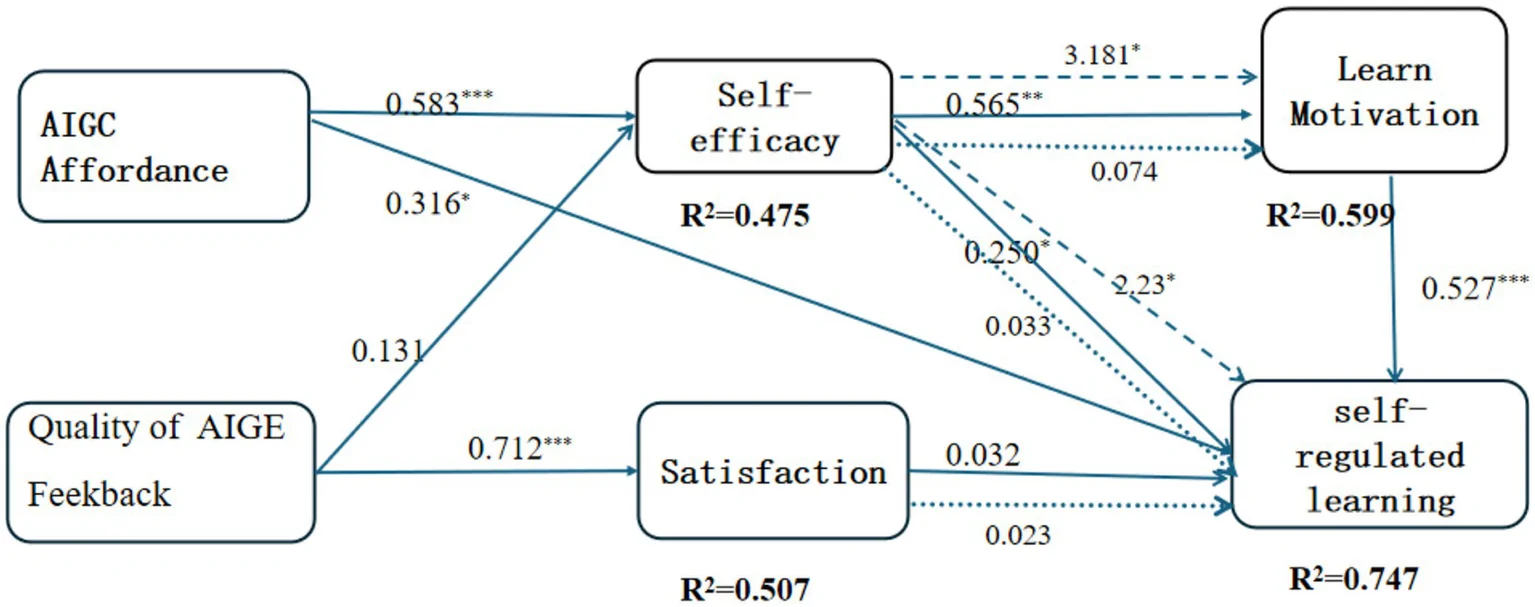
Results of research model. ^*^p < 0.05; ^**^p < 0.01; ^***^p < 0.001.

The mediating hypotheses (H6–H12) were further examined using bootstrapping based indirect effect tests. Table 8 and Figure 2 summarize the structural paths and hypothesis-testing results.

**Table 8:** Hypotheses testing results.

Hypothesis	Path coefficient (*β*)	Results
H1a: AIGC affordance → AIGC self-efficacy	0.583	Supported
H1b: AIGC affordance → Student self-regulated learning	0.316	Supported
H2a: AIGC quality assessment Feedback → Satisfaction	0.712	Supported
H2b: AIGC quality assessment Feedback → AIGC self-efficacy	0.131	No supported
H3a: AIGC self-efficacy → Learn motivation	0.565	Supported
H3b: AIGC self-efficacy → Student self-regulated learning	0.250	Supported
H4: Learn motivation → Student self-regulated learning	0.527	Supported
H5: Satisfaction → Student self-regulated learning	0.032	No supported
H6: Affordance → Self-efficacy → Learn motivation	0.329	Supported
H7: Affordance → Self-efficacy → Student self-regulated learning	0.145	Supported
H8: AIGC Quality assessment feedback → Self-efficacy → Learn motivation	0.074	No supported
H9: AIGC Quality assessment feedback → Self-efficacy → Student self-regulated learning	0.033	No supported
H10: AIGC Quality assessment feedback → Satisfaction → Student self-regulated learning	0.023	No Supported
H11: AIGC affordance → Self-efficacy → Learn motivation → Student self-regulated learning	0.173	Supported
H12: AIGC Quality assessment feedback → Self-efficacy → Learn motivation → Student self-regulated learning	0.039	No supported

### 4.3 Mediation test

To examine the mediating roles of AIGC self-efficacy and satisfaction, we employed Hair et al.’s (2022) bootstrapping analysis with 10,000 subsamples and corrected confidence intervals (95% CI), following the methodology outlined by Fang (2019). According to Fang (2019), an indirect mediation effect is considered significant if the corrected confidence interval does not include zero. As presented in Table 9, the results indicate AIGC self-efficacy mediates the influences of support for AIGC affordance on learn motivation and student self-regulated learning.

**Table 9:** Indirect and mediating effects.

Direct effect			Indirect effect					
						Bootstrap 95% CI		
Path	Coeffi-cient	*t*-value	Path	Coeffi-cient	*t*-value	Percentile	Bias corrected	Effect of mediation
AFS → LM	−0.227	1.278	AFS → SEF → LM	0.329	3.181^*^	[0.075:0.479]	[0.164:0.561]	Significant
AFS → SRL	0.316	2.198^*^	AFS → SEF → SRL	0.145	2.23^*^	[−0.014:0.242]	[0.041:0.292]	Significant
			AFS → LM → SRL	−0.120	1.216	[−0.276:0.105]	[−0.364:0.027]	No significant
			AFS → SEF → LM → SRL	0.173	2.361^*^	[0.041:0.408]	[0.058:0.367]	Significant
QAF → LM	0.500	2.785^*^	QAF → SEF → LM	0.074	0.280	[−0.045:0.323]	[−0.008:0.298]	No significant
QAF → SRL	−0.153	0.321	QAF → SEF → SRL	0.033	1.103	[−0.019:0.094]	[−0.004:0.125]	No significant
			QAF → SAT → SRL	0.023	0.316	[−0.019:0.094]	[−0.124:0.165]	No significant
			QAF → LM → SRL	0.264	2.770^**^	[0.049:0.420]	[0.117:0.504]	Significant
			QAF → SEF → LM → SRL	0.039	1.018	[0.002:0.459]	[−0.000:0.192]	No significant

AFS, AIGC affordance; LM, Learn Motivation; QAF, AIGC Quality Assessment Feedback; SAT, Satisfaction; SRL, Student Self-Regulated Learning; SEF, AIGC Self-efficacy. *p < 0.05, **p < 0.01, ***p < 0.001.

Additionally, the results also reveal that the learning motivation mediates the influences of support for AIGC affordance and AIGC self-efficacy on learn motivation. Thus, both AIGC self-efficacy and learn motivation have significant mediation effects in this research model.

## 5 Discussion

This study clarifies how AIGC supports student learning within industry– education integration (IEI) by linking technology perceptions to self-regulated learning (SRL) through efficacy and motivation pathways. Consistent with research on generative AI in education (e.g., Huang et al., 2024a, 2024b), perceived AIGC affordance was strongly associated with higher AIGC self-efficacy, which in turn increased learning motivation and SRL. These results extend IEI scholarship by showing that AIGC is not only a functional tool for completing tasks but also a psychological enabler that helps students plan, monitor, and adjust their learning.

Two non-significant links are theoretically informative. H2b indicates students may value actionable guidance over positive feedback: accurate yet generic explanations produce transient satisfaction without strategic knowledge. H5 suggests satisfaction may reflect cognitive offloading (Risko and Gilbert, 2016) rather than regulatory internalization, with students experiencing successful outsourcing, not active mastery. This aligns with Reeve and Cheon (2021) and Tripon (2021): autonomy-supportive guidance that designs student autonomy outperforms directive support in fostering sustained reengagement. Both imply intentionally incomplete feedback and verification rubrics may better scaffold SRL than seamless experiences.

Qualitative findings reveal active boundary conditions on psychological processes. Curriculum-industry misalignment reduces task authenticity, limiting genuine mastery opportunities and constraining self-efficacy to contingent confidence. Weak enterprise engagement inflates perceived feedback quality without skill development—without practitioner mentors to challenge AI outputs, students’ successful interactions reflect epistemic overconfidence. Drawing on Tripon (2021), we argue that supportive mentors use quality educational relationships to stimulate students’ involvement in their own learning and design autonomy. This implies that AIGC feedback alone is insufficient; it is the autonomy-supportive guidance from mentors that transforms positive AI experiences into genuine self-efficacy and self-regulated learning. Where enterprise engagement is weak and mentors fail to provide such autonomy support, even high-quality AIGC feedback cannot activate the regulatory pathway (H2b, H5). Limited project diversity narrows AIGC applications, diminishing autonomy and competence satisfaction (Ryan and Deci, 2021). Thus, quantitative paths are necessary but insufficient: affordance activates SRL only with authentic institutional support.

Limitations and future directions. The proposed serial mediation rests on theoretical temporal logic rather than empirical sequencing. Alternative causal configurations remain plausible: high-SRL students may selectively perceive affordances (reverse causality), or self-efficacy and motivation may reciprocally reinforce. Cross-sectional data preclude strong causal claims; longitudinal or experimental designs—such as manipulating affordance salience through structured versus open-ended AIGC interfaces—are needed to adjudicate these possibilities.

Implications follow for policy and teaching. At the program level, co-development with enterprises should prioritize timely competency mapping, clearer expectations for mentors, and stable communication platforms. At the classroom level, instructors can use AIGC to scaffold SRL by embedding prompt-and-reflect routines, feedback literacy activities, and verification rubrics that require students to justify and triangulate AI outputs rather than accept them uncritically. Finally, future work could track SRL processes longitudinally and examine when AIGC use shifts from supportive scaffolding to dependence across different IEI disciplines and task types.

## 6 Conclusion

Drawing on survey and interview evidence from an IEI pilot context in Guangzhou, this study advances evaluation of industry–education integration by explaining how AIGC-related perceptions translate into students’ self-regulated learning (SRL). The results indicate that AIGC affordance strengthens AIGC self-efficacy, which increases learning motivation and, in turn, SRL. In other words, AIGC contributes most when it builds students’ confidence and persistence to regulate their own learning, rather than when it simply delivers answers.

At the same time, AIGC assessment-feedback quality primarily improves satisfaction, and satisfaction does not directly predict SRL. This finding cautions against equating “good AI experience” with deep learning: without motivational activation, even high-quality feedback may remain a surface-level benefit. Qualitative findings further show that IEI effectiveness is constrained by curriculum–industry misalignment, limited project diversity, and uneven enterprise engagement, reinforcing the need to evaluate IEI with indicators of authentic application and learning regulation.

Practically, IEI programs should (a) strengthen enterprise co-development and mentorship mechanisms, (b) update curricula based on current competency needs, and (c) embed SRL-oriented AIGC pedagogy (prompt scaffolds, verification routines, and feedback literacy). Future research could use longitudinal or experimental designs to test causal effects of AIGC-enabled interventions on SRL and to identify boundary conditions (e.g., discipline, task complexity, and students’ prior AI literacy).

## Data Availability

The raw data supporting the conclusions of this article will be made available by the authors, without undue reservation.
